# Clinical manifestations of 42 Moroccan patients with chronic granulomatous disease

**DOI:** 10.5339/qmj.2025.14

**Published:** 2025-03-21

**Authors:** Assma Dably, Ibtihal Benhssaein, Jalila El Bakkouri, Asmaa Drissi Bourhanbour, Leila Jeddane, Ahmed Aziz Bousfiha, Fatima Ailal

**Affiliations:** ^1^Laboratory of Clinical Immunology, Inflammation and Allergy LICIA, Faculty of Medicine and Pharmacy, Hassan II University, Casablanca, Morocco; ^2^Department of pediatric infectious and immunological diseases, Abderrahim El Harouchi Children's Hospital, University Hospital Center Ibn Rochd, Casablanca, Morocco; ^3^Laboratory of Immunology, University Hospital Center Ibn Rochd, Casablanca, Morocco; ^4^National Reference Laboratory, Cheikh Khalifa International University Hospital, Casablanca, Morocco *Correspondence: Assma Dably. Email: asmaadably@gmail.com

**Keywords:** Primary immunodeficiency, innate error, chronic granulomatous disease, aspergillosis, DHR test

## Abstract

**Background:**

Chronic granulomatous disease (CGD) is a primary immunodeficiency due to alterations in the oxidative metabolism of phagocytic cells. This condition is characterized by serious and recurrent infections caused by pyogenic bacteria, particularly *Staphylococcus aureus*, and fungal pathogens such as *Aspergillus*. These infections are associated with granuloma formation and inflammatory manifestations.The aim of our study was to report the clinical characteristics, microbiological aspects and outcomes, and prognosis of a cohort comprising 42 Moroccan patients suffering from CGD.

**Methods:**

A total of 42 patients were diagnosed for family history, consanguinity, and both clinical and laboratory findings.The diagnosis was confirmed by assessing neutrophil oxidative burst activity, using either the nitroblue tetrazolium (NBT) test or the dihydrorhodamine (DHR) test.

**Results:**

The cohort comprised children from 34 different families, including 12 siblings. The age of onset ranged from 4 days to 13 years, with the diagnosis being established between the ages of 25 days and 13 years. The predominant clinical manifestations were skin infections, lymphadenopathy, pneumonia, BCGitis, liver abscess, pulmonary aspergillosis, and inflammatory colitis. The most frequently isolated germs were *Aspergillus*, *Serratia*, and *Staphylococcus*. Among the total of 42 patients, 17 fatalities occurred, with aspergillosis being identified as the primary cause of their deaths.

**Conclusions:**

In this study, the clinical characteristics and isolated microorganisms correspond to the pathogens known to be important in CGD. Lung infections represent the most prevalent complication and significantly contribute to high mortality rates, particularly in the case of *Aspergillus* pneumonia, which is known for its tendency to disseminate. Additionally, BCGitis has been frequently observed in countries where the BCG (Bacille Calmette–Guérin) vaccination is routinely administered. Enterocolitis emerges as the most common inflammatory complication in clinical settings. Unfortunately, CGD remains largely unknown in Morocco, highlighting the urgent need to raise awareness among doctors. This increased awareness could facilitate early diagnosis and improve patient prognosis.

## Introduction

Chronic granulomatous disease (CGD) is a rare hereditary primary immunodeficiency affecting innate immunity, stemming from a defect in phagocyte bactericidal activity secondary to mutations in the genes encoding NADPH oxidase subunits. CGD is clinically manifested by life-threatening bacterial and aspergillosis infections of the respiratory tract, skin, lymph nodes, and liver.

According to the 2022 phenotypic classification of primary immunodeficiencies established by the International Union of Immunological Societies, CGD is classified in the group of congenital phagocyte defects, specifically in the subgroup of functional defects.^1^

Approximately 1/250,000 live births worldwide is affected by this disease, with onset at a very early age (less than 2 years).^2^

The incidence rates are almost identical in all ethnic and racial groups. However, in regions where consanguineous marriages are common (the MENA: middle east North of Africa region), the autosomal recessive (AR-CGD) form is more common than the X-linked recessive (XL-CGD) form, leading to higher overall incidence rates compared to Western countries, where the AR subtype is less common than the XL-CGD subtype.

CGD patients have suffered repeated episodes of infection since childhood. The disease exhibits significant heterogeneity, as evidenced by the diverse range of affected organs in both children and adults. The infections are primarily caused by microorganisms, including fungi and “catalase-positive” bacteria.^3,4^

CGD is characterized by the inability of phagocytic cells to produce the oxygen-active metabolites for the effective elimination of pathogens (fungi and certain bacteria). Although these microorganisms are typically engulfed by phagocytes, they are able to survive within these cells. In addition, the resulting infectious foci stimulate granuloma formation, partly due to the release and persistence of chemoattractants that require reactive forms of oxygen for their degradation.^4,5^

CGD is a rare chronic hereditary disease that warrants thorough investigation and recognition by both health professionals and the general public.^6^ Moreover, it is posited that CGD is very common in Morocco, attributable to ethnic characteristics and a high rate of consanguineous marriages. The lack of awareness among medical practitioners may result in this pathology being overlooked in numerous patients, overlapping with other differential diagnoses.

This article presents a cohort of patients with CGD in Morocco, focusing on their clinical and microbiological characteristics, as well as the evolution and prognosis of the disease.

## Methods

The present study was a retrospective descriptive analysis of patients with CGD who received follow-up care at the clinical immunology unit at the Abderrahim El Harouchi Children's Hospital within the Ibn Rochd University Hospital in Casablanca, Morocco from 1997 to 2023.

A total of 42 patients were identified over a period of 26 years, with data collected through an examination of medical records.

During the first 17 years of the study (1997–2014), only 12 cases of CGD were diagnosed. However, from 2014 to 2023, the number of CGD patients increased by 30 cases, due to the enhanced knowledge and awareness among medical professionals regarding the disease, as well as improved access to diagnostic testing at our center.

The study received ethical approval from the Ibn Rochd University Hospital ethics committee.

The diagnosis of CGD was based on clinical data and subsequently validated through functional tests of the patients’ neutrophils.

Inclusion criteria were unusual and recurrent infectious episodes, such as adenopathy, pulmonary aspergillosis, local BCGitis, and liver abscesses, at a very early age, along with exaggerated inflammatory responses. Initial evaluations were normal, including CBC, immunoglobulin levels, lymphocyte subpopulation tests, and functional neutrophil tests (nitroblue tetrazolium (NBT) and dihydrorhodamine (DHR) tests) that confirmed the disease.

Exclusion criteria are HIV infection and the presence of neutropenia.

The investigation focused on older patients who underwent the NBT test, with subsequent confirmation of their diagnosis through the DHR test beginning in 2014. After 2014, the diagnosis was based solely on the DHR test conducted via flow cytometry. Blood samples were analyzed using the commercial Phagoburst reagent kit (Glycotope Biotechnology, Germany) following the manufacturer's protocol. This kit contains three stimulants: *E.coli*, PMA: Phorbol myristate acetate, and FMLP: formyl-methionyl-leucyl-phenylalanine. Samples were analyzed on a BD FACSDiva flow cytometer software version 8.0 (San Jose, CA).

## Results

### Demographics and family history

The cohort consisted of 85.7% males, with five patients being siblings (Table 1). Additionally, 40.48% of the patients had consanguineous parents, categorized into 13 different families (Table 1).

**Figure 1.**
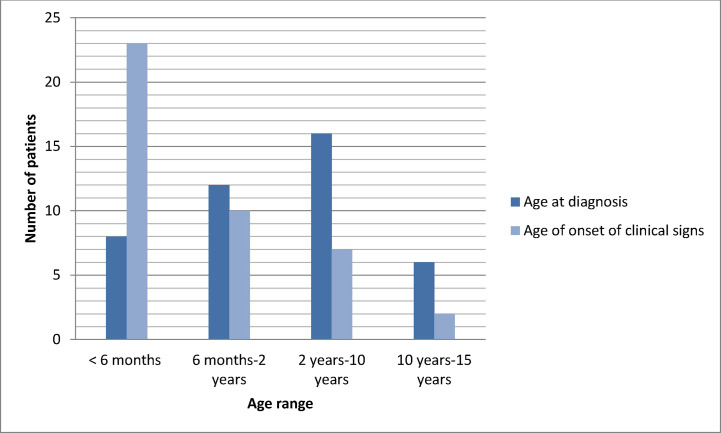
Distribution of CGD patients according to the age of onset of clinical signs and the age at diagnosis.

The mean age at onset of symptoms and at diagnosis was 1.62 years (ranging from 0.02 to 13 years) and 3.74 years (ranging from 0.07 to 13 years), respectively. Notably, 78.57% of the patients exhibited symptoms before reaching 2 years of age, while 52.3% of cases were diagnosed after the age of 2 (Figure 1).

### Clinical manifestations

All patients in our study received a diagnosis following the onset of infections. The most commonly observed infections were those located in the skin and mucous membranes, affecting 57.14% of the patients. Among these patients, 29.2% developed localized skin abscesses on the hands, feet, and face, 25% had perianal abscesses, 12.5% had pyoderma, 12.5% had oral aphthous ulcers, 12.5% had oral thrush, and 4.2% had ecthyma gangrenosum (Table 2).

Respiratory infections were observed in 54.8% of the patients, 34.8% had pulmonary aspergillosis, 47.8% had pneumonia, and 13% had purulent pleurisy. Additionally, one case was attributed to *Pseudomonas*, another to *Shewanella*, and one patient had a pulmonary abscess (Table 2).

In the study, lymphadenopathy was identified in 42.86% of the patients, predominantly in the cervical region. Specifically, 50% of these cases were classified as cervical lymphadenopathy (Table 2).

BCGitis was observed in 23.81% of the patients, with 60% presenting as localized BCGitis and 40% as locoregional BCGitis (Table 2).

Hepatic abscesses were reported in 9.52% of the patients, with one case revealing the presence of *Aspergillus* isolated from the pus.

Additionally, 16.67% of the patients had osteoarticular infections, including 57.1% diagnosed with osteitis. Among these, 50% had sternal osteitis caused by *Aspergillus*, while 42.9% were diagnosed with osteomyelitis, including one case of chronic osteomyelitis due to both *Staphylococcus* and *Aspergillus*.

Septicemia was observed in 16.67% of the patients, including 14.3% with *Pseudomonas* septicemia, which was complicated by leg and finger amputations, and another 14.3% with septicemia caused by *Serratia marcescens*.

Central nervous system infections were observed in 14.28% of the patients, with specific conditions including cerebral abscesses in 33.3% of cases, meningitis in 16.7% of cases, meningoencephalitis in 16.7% of cases due to *Trichosporum*, and cerebral aspergillosis in 33.3% of cases.

Furthermore, 23.8% of the patients had digestive manifestations, with diarrhea occurring in 50% of cases, primarily caused by *Salmonella*, and 50% presenting with inflammatory colitis. Additionally, hemophagocytic lymphohistiocytosis (HLH) was noted in 4.7% of the patients, while lupus and pyoderma gangrenosum were each observed in 2.3% of the patients.

### Functional test

**Figure 2.**
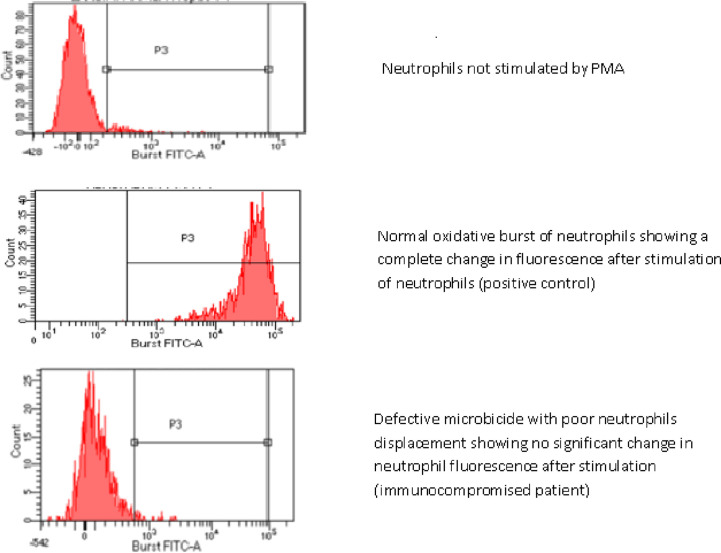
Exploration of oxidative microbicidal activity by flow cytometry (DHR test) in a patient with CGD.

All patients diagnosed by the DHR test showed an abnormal neutrophil response to PMA (Figure 2).

### Treatment

All patients were placed on prophylactic treatment with trimethoprim-sulfamethoxazole at a dosage of 25 mg/kg/day and itraconazole at 10 mg/kg/day immediately following diagnosis. No patient underwent a hematopoietic stem cell allograft. Each infectious episode was initially treated empirically with antibiotics, subsequently tailored according to the antibiogram results.

Aspergillus infections were first treated with intravenous voriconazole, followed by an extended course of oral therapy until there was clinical improvement and clearance of antigenemia and *Aspergillus* serology. In contrast to refractory cases, amphotericin B was used.

Patients with BCGitis were treated with rifampicin, INH, and ethambutol for locoregional BCGitis, while those with local BCGitis were treated with a dual therapy based on rifampicin and INH.

Surgical interventions were performed in only two cases: evacuation of a liver abscess and excision of a large axillary adenopathy associated with locoregional BCGitis.

Inflammatory colitis was initially treated with Pentasa, and in cases of exacerbation, corticosteroids were administered, along with immunosuppressants such as Imurel, with biotherapy being used in certain cases.

### Evolution

In terms of patient outcomes, 33.3% of cases achieved satisfactory results with treatment alone, exhibiting no signs of infection.

Clinical follow-up, particularly through the measurement of calprotectin levels, indicated that 11.9% of the patients exhibited gastrointestinal involvement, particularly inflammatory colitis.

Among the cohort of 42 patients, the mortality rate was 40.48%. The causes of death included pulmonary and cerebral aspergillosis in 17.6% of cases, infections (with septicemia accounting for 71.4%) in 41.2% of cases, HLH in 5.9% of cases, and inflammatory colitis in 5.9% of cases.

## Discussion

CGD is a rare genetic primary immunodeficiency resulting from a defect in the oxidative metabolism of neutrophils, leading to susceptibility to severe and recurrent life-threatening bacterial and fungal infections.^13^

The general prognosis of this pathology has improved significantly due to the therapeutic advances, enabling patients to prevent infection risks through good skin hygiene practices and the lifelong administration of antifungals and antibiotics.

Upon diagnosis of the disease, treatment is based on three principles: first, the administration of lifelong antimicrobial prophylaxis; second, the necessity for early diagnosis at the onset of infection; and finally, the requirement for vigorous treatment of infectious and inflammatory complications.^14^

In the past, children with CGD had a high rate of mortality. Today, the disease is manageable, resulting in a high survival rate. Patients can typically expect to live approximately 40 years, especially adhering to long-term prophylactic antimicrobial therapy.^14^ In Central Anatolia, the survival rate following hematopoietic stem cell transplantation is 87.5%.^15^

Morbidity and mortality may be influenced by chronic inflammation. CGD is histologically characterized by the presence of inflammatory granulomas. These granulomas are formed by the interaction of macrophages, monocytes, T lymphocytes, and antigens through specific receptors and cytokines, which may explain the chronic nature of the inflammatory response. Inflammatory manifestations can affect multiple organs, such as the urinary tract, where stenosing granulomatous inflammatory lesions may occur.^14^

This study represents a cohort of 42 Moroccan patients with CGD. The majority of these patients seem to be affected by the AR form of the disease, given the high rate of consanguineous marriages in Morocco (22.79%).^16^ This finding may be comparable with data from Egypt, with a consanguinity rate of 81.5% relative to 76.3% of AR forms,^7^ and from Turkey, with a consanguinity rate of 43.82% corresponding to 55% of AR forms.^11^

The mean age at onset of clinical signs was 1.62 years, consistent with findings from studies conducted in Egypt (0.58 years), France (0.94 years), and Mexico (0.25 years),^7,9,10^ all indicating that initial clinical manifestations of the disease occurred during the first year of life.

The mean age at diagnosis in our cohort was 3.74 years, which is similar to data from several countries such as Iran (3.9 years), Egypt (4 years), and Turkey (4.2 years).^7,11,17^ This difference may be due to a lack of awareness of the disease. However, the mean age at diagnosis was lower in studies from France (2.52 years) and Mexico (1.17 years).^9,10^

Skin regions are exposed to many pathogenic organisms and contain a large population of reticuloendothelial cells, which are important sites of infection in CGD patients. In our study, the most common clinical manifestation observed was skin involvement (57.14%), followed by lung involvement (52.38%) and lymph nodes (42.86%). These findings are consistent with an Iranian study that reported 53% of patients with skin infections, 50% with pneumonia, and 29% with lymphadenopathy.^17^

On the contrary, other studies have reported that respiratory tract infections are the most common manifestation, followed by skin involvement, as observed in European and Egyptian cohorts.^7,12^ In addition, both a French study and a Turkish study identified pulmonary involvement as the main infection, followed by adenopathy.^9,11^

Microorganisms isolated exhibit significant variation across different countries. Studies conducted in Mexico and the United States identified *Staphylococcus* as the most frequently detected pathogen.^8,10^ Conversely, research from Egypt highlighted *Aspergillus* as the most frequently isolated microorganism, followed by *Mycobacteria* sp. and *Staphylococcus*.^7^ Furthermore, the Turkish study found *Aspergillus* to be the most frequently detected pathogen, followed by *Staphylococcus* and *Serratia*.^11^ In a French cohort, *Aspergillus* was reported as the primary microorganism associated with serious invasive infections, followed by *Staphylococcus* and *Serratia*.^9^

Similar to global trends, the microorganisms present in our cohort are predominantly *Aspergillus*, followed by *Serratia* and *Klebsiella. Aspergillus* was identified in 16.67% of the 22 respiratory cases, 33.3% of the six cerebral cases, 50% of the four osteitis cases, 33.3% of the three osteomyelitis cases, and 25% of the four liver abscess cases (Table 3). The predominance of *Aspergillus* in our findings may be due to high exposure to environmental fungal species.

The administration of the Bacille Calmette–Guérin (BCG) vaccine at birth is mandatory. However, BCGitis represents a serious risk for immunocompromised children, especially those with CGD.^18^

In our study, 23.8% of the vaccinated patients developed BCGitis. To avoid complications, this vaccine is contraindicated in cases of primary immune deficiencies, including phagocytic cell deficiencies such as CGD.

In addition to respiratory infectious manifestations, bronchiectasis was reported in only one patient. This is in line with findings from a Sri Lankan study, which also reported only one case of bronchiectasis.^2^

Conversely, CGD may lead to inflammatory bowel disease, exhibiting histological and endoscopic aspects similar to those found in Crohn's disease. In our cohort, gastrointestinal manifestations, such as inflammatory colitis, were observed in 11.9% of patients. An Iranian study indicated that 42% of patients were diagnosed with gastrointestinal manifestations, with diarrhea being the most common complication.^17^

The diagnosis of CGD in the present study was primarily confirmed through the NBT slide test. Currently, this test is seldom used due to its subjectivity, low sensitivity, and the need for significant expertise. As a result, it has been replaced by the DHR test conducted via flow cytometry, which has been applied to all living patients in Morocco since its validation. Unlike the qualitative NBT test, the DHR test is highly sensitive and specific, enabling a quantitative assessment of NADPH oxidase enzyme activity and the detection of XL-CGD carrier status in mothers.^19^

The mortality rate observed in our cohort (40.48%) is similar to that reported in Sri Lanka (38.4%),^2^ Mexico (39.8%),^10^ and Tunisia (29.26%).^20^ The notable increase in the number of deaths in our study can be attributed mainly to the absence of hematopoietic stem cell transplantation for this condition in Morocco. In contrast, lower mortality rates were reported in France (12.5%)^9^ and the Unites States (15.3%)^8^ (Table 4).

## Conclusion

The cohort of Moroccan patients with CGD presented common clinical manifestations associated with the condition. The skin was the most commonly affected site of infection, followed by the lungs. Despite antimicrobial prophylactic treatment, most patients experienced severe recurrent infections and suffered from serious, potentially life-threatening bacterial and fungal infections, particularly involving *Aspergillus* and *Serratia*. Therefore, the administration of the BCG vaccine to infants presenting with this condition is not recommended.

The mortality rate observed in our cohort is considerably high compared to larger cohorts from Western countries and the Americas. In Morocco, treatment approach primarily relies on immediate antibacterial and anti-*Aspergillus* prophylaxis to prevent the occurrence of severe and sometimes challenging infections, particularly invasive aspergillosis. It is imperative to enhance awareness among physicians regarding the early diagnosis of this primary immunodeficiency, as the symptoms of this disease are diverse and highly indicative.

### Competing interests

The authors have no conflicts of interest to declare.

## Figures and Tables

**Table 1.** Clinical and demographic data of CGD patients.

Patients	Gender		Consanguinity	Death	Mean age of onset of clinical signs	Mean age at diagnosis

42	M	F	17	17	1.62	3.74

	36	6				

Main clinical signs				Mucocutaneous infections (57.14%)Respiratory infections (54.8%)Adenopathies (42.86%)BCGitis (23.8%)Osteoarticular infections (16.67%)Sepsis (16.67%)Neurological disorders (14.28%)Inflammatory colitis (11.9%)Splenomegaly (11.9%)Liver abscess (9.5%)		

All clinical manifestations						

- Hepatic abscess, perianal abscess, *Klebsiella* inguinal adenopathy- Aspergillus sternal osteitis, chronic osteomyelitis caused by *Staphylococcus*and *Aspergillus*- *Salmonella*diarrhea, HLH, bilateral Bronchiectasis, cerebral aspergillosis, fistulized adenopathy- Inflammatory colitis, thigh abscess, psoas abscess- Sepsis, oral thrush, *Serratia* adenopathy, facial abscesses, oral aphthosis- Local BCGitis, skin abscess, *Staphylococcus*cervical adenopathy- Pulmonary aspergillosis, locoregional BCGitis, liver and brain abscesses- *Trichosporum* meningoencephalitis, *Aspergillus* osteitis of the femoral head, right basal DDB- Lupus, pneumopathy, cervical adenopathy, diarrheal septicemia- *Pseudomonas*septicemia complicated by the amputation of a leg and fingers- Left maxillary adenopathy, omphalitis, oral candidiasis, ecthyma gangrenosum- Infectious pneumonia, *Serratia* fistulized cervical adenitis, vertebral osteitis- *Serratia marcescens* septicemia, meningitis, splenomegaly, *Pseudomonas* purulent pleurisy- Excavated *Streptococcus viridans* and *Candida tropicalis* pneumonia- *Streptococcus group A* adenopathy, *Serratia marcescens* adenopathy- Bacterial osteitis, scalp abscess, febrile diarrhea, right cervical adenophlegmon- Recurrent *Serratia* cervical adenophlegmon, orbital cellulitis, facial boils- *Klebsiella pneumoniae* adenophlegmon, right inguinal cervical submandibular tuberculous- Bilateral pneumopathy, scalp pyoderma, inguinal adenopathy, hepatic aspergillosis- Pyoderma gangrenosum, *Klebsiella pneumonia*, cervical and pulmonary aspergillosis- Costal osteitis, recurrent pyoderma, hepatosplenomegaly, inguinal adenopathy- *Serratia* and *Klebsiella* skin abscesses, bronchopneumonia, *Shewanella* pleurisy						


**Table 2.** Clinical manifestations of chronic granulomatous disease.

Clinical signs	Examples

A, B, and C: Pulmonary and liver aspergillosis with lumbar extension	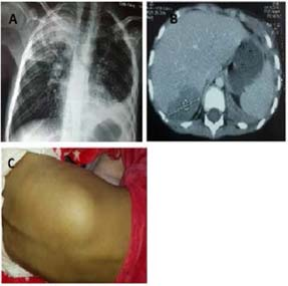

D and E: BCGitis and lymphadenopathy due to *Staphylococcus aureus*	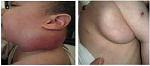

F: Skin infection due to *Serratia*	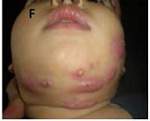

G: Cerebral aspergillosis	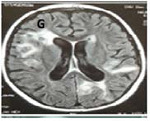


**Table 3.** Proportions of pathogenic microorganisms identified in our patient cohort.

Gram-positive cocci (*n*=9)	Gram-negative bacilli (*n*=26)	Fungi (*n*=26)

- 5 *Staphylococcus* (55.5%) - 2 *Enterococcus faecium* (22.2%) - 2 *Streptococcus group A* (22.2%)	- 8 *Serratia marcescens* (30.8%) - 1 *Serratia liquefaciens* (3.8%) - 7 *Klebsiella pneumoniae* (26.9%) - 3 *Salmonella typhimurium* (11.5%) - 2 *Pseudomonas* (7.7%) - 1 *Enterobacter aerogenes* (3.8%) - 1 *Acinetobacter baumannii* (3.8%) - 1 *Shewanella* spp. (3.8%) - 1 *Escherichia coli* (3.8%)	- 10 *Aspergillus* spp. (62.5%) - 3 *Candida albicans* (18.75%) - 1 *Candida dubliniensis* (6.25%) - 1 *Candida tropicalis* (6,25%) - 1 *Trichosporum* (6.25%)


**Table 4.** Demographic comparison of CGD patients.

Country	Number of patients	Gender		Consanguinity	Mortality

		Male	Female		

Morocco	42	36	6	17 (40.48%)	17 (40.48%)

Egypt^7^	173	107	66	141 (81.5%)	54 (31.4%)

USA^8^	27	23	4	5 (18.5%)	4 (15.3%)

Sri Lanka^2^	12	10	2	6 (46%)	4 (38.4%)

France^9^	80	71	9	-	10 (12.5%)

Mexico^10^	93	82	11	10 (10.75%)	37 (39.8%)

Turkey^11^	89	64	25	39 (43.82%)	9 (10.11%)

Europe^12^	429	351	78	-	84 (20%)

